# First-principles Hubbard parameters with automated and reproducible workflows

**DOI:** 10.1038/s41524-025-01685-4

**Published:** 2025-06-16

**Authors:** Lorenzo Bastonero, Cristiano Malica, Eric Macke, Marnik Bercx, Sebastiaan Huber, Iurii Timrov, Nicola Marzari

**Affiliations:** 1 U Bremen Excellence Chair, Bremen Center for Computational Materials Science, and MAPEX Center for Materials and Processes, University of Bremen, D-28359 Bremen, Germany; 2 PSI Center for Scientific Computing, Theory, and Data, and National Centre for Computational Design and Discovery of Novel Materials (MARVEL), 5232 Villigen PSI, Switzerland; 3 Theory and Simulation of Materials (THEOS), and National Centre for Computational Design and Discovery of Novel Materials (MARVEL), École Polytechnique Fédérale de Lausanne (EPFL), CH-1015 Lausanne, Switzerland

**Keywords:** Electronic properties and materials, Batteries, Condensed-matter physics

## Abstract

We introduce an automated, flexible framework (aiida-hubbard) to self-consistently calculate Hubbard *U* and *V* parameters from first-principles. By leveraging density-functional perturbation theory, the computation of the Hubbard parameters is efficiently parallelized using multiple concurrent and inexpensive primitive cell calculations. Furthermore, the intersite *V* parameters are defined on-the-fly during the iterative procedure to account for atomic relaxations and diverse coordination environments. We devise a novel, code-agnostic data structure to store Hubbard related information together with the atomistic structure, to enhance the reproducibility of Hubbard-corrected calculations. We demonstrate the scalability and reliability of the framework by computing in high-throughput fashion the self-consistent onsite *U* and intersite *V* parameters for 115 Li-containing bulk solids with up to 32 atoms in the unit cell. Our analysis of the Hubbard parameters calculated reveals a significant correlation of the onsite *U* values on the oxidation state and coordination environment of the atom on which the Hubbard manifold is centered, while intersite *V* values exhibit a general decay with increasing interatomic distance. We find, e.g., that the numerical values of *U* for the 3d orbitals of Fe and Mn can vary up to 3 eV and 6 eV, respectively; their distribution is characterized by typical shifts of about 0.5 eV and 1.0 eV upon change in oxidation state, or local coordination environment. For the intersite *V* a narrower spread is found, with values ranging between 0.2 eV and 1.6 eV when considering transition metal and oxygen interactions. This framework paves the way for the exploration of redox materials chemistry and high-throughput screening of *d* and *f* compounds across diverse research areas, including the discovery and design of novel energy storage materials, as well as other technologically-relevant applications.

## Introduction

Density-functional theory^1,2^ (DFT) has become a workhorse of computational condensed-matter physics, chemistry, and materials science^3^. Its long-standing success is based on a favorable balance of accuracy and computational efficiency that is achieved by mapping the complex many-body problem of interacting electrons onto an auxiliary system of non-interacting particles moving in an effective potential. The primary challenge in DFT applications lies in the exchange-correlation (xc) functional, whose exact analytical form is unknown and must therefore be approximated. Among the numerous xc functionals proposed, the local-density approximation (LDA) and the generalized-gradient approximation (GGA)^4^ are the simplest local/semi-local choices, mainly for efficiency reasons. However, despite their successful applications to a large variety of systems, these functionals have proven much less adequate for the treatment of transition-metal (TM) and rare-earth (RE) compounds. These issues originate from electron self-interaction errors (SIEs)^5,6^, which particularly plague the description of partially occupied and localized *d* and *f* states. Different xc functional flavors have been proposed to cure this flaw: Hubbard corrections to DFT^7–13^, meta-GGA functionals, such as SCAN and its variants^14–16^ (as well as SCAN+*U*^17–20^) and hybrid functionals (e.g., PBE0^21^ and HSE06^22,23^), to name a few.

In Hubbard-corrected DFT^7–10^, one or several corrective terms are added to the base DFT xc functional (typically LDA or GGA), whose strength is gauged by the numerical values of the associated *Hubbard parameters*. The most widespread formulations include the “on-site” *U* terms, which promote localization of electrons on atomic sites; “inter-site” *V* terms stabilizing states between two atoms^11^; and Hund’s *J* terms that account for the opposite-spin interactions within a given shell^24–26^. The first-principles determination of these parameters can be achieved by recognizing^27,28^ that the rotationally invariant formulation of DFT+*U* provides a natural correction for the spurious curvature of (semi)local functionals, by a removal of a quadratic term and the addition of a linear one. This heuristic connection, valid in the weak coupling limit between the target Hubbard manifolds and the rest of the electron bath, allows to calculate the Hubbard parameters from first-principles by means of the linear response of the occupation matrices using constrained DFT (LR-cDFT)^28^. Recently, its reformulation in terms of density-functional perturbation theory (DFPT)^29,30^ boosted its success owing to the replacement of expensive supercells by a computationally less demanding primitive cell with monochromatic perturbations. Other strategies for computing Hubbard parameters have also been proposed, including Hartree-Fock based methods^12,13,31–34^ and the constrained random phase approximation (cRPA)^35–38^. Although Hubbard corrections were originally developed to improve the description of strongly correlated materials (typically involving *d* or *f* elements), their success primarily derives from the *U* correction’s ability to enforce piece-wise linearity and remove electronic self-interaction^39^. This mechanism alleviates the overstabilization of fractional occupations in standard (semi)local functionals—a problem that arises from the incomplete cancellation between the xc functional and the Hartree term, especially for localized electrons. This improvement is further evidenced by the marked qualitative and quantitative enhancements observed in the electronic structure of molecular systems containing a single transition metal atom when DFT+*U* is applied^39,40^. In this light, the DFT+*U* correction serves as a self-interaction correction.

These well-established methods provide frameworks to compute Hubbard parameters, but their outcome strongly depends on the ground state being perturbed. In other words, Hubbard parameters computed for an uncorrected DFT ground state (i.e., *U* = 0) may differ significantly from those obtained for a corrected one (i.e., *U* > 0). Obtaining a stable set of Hubbard parameters thus requires a self-consistent cycle in which a new set of Hubbard parameters is evaluated from a corrected DFT+*U*(+*V*) ground state that was determined using the Hubbard parameters from the previous step. This cycle can also be combined with structural optimizations^30,41–43^, thus allowing for mutual consistency between the ionic and electronic DFT+*U*(+*V*) ground states. Hubbard *U* and *V* parameters determined using this procedure often lead to significant improvements in electronic structure properties, such as accurate digital changes in oxidation states^44^, even in first-principles molecular dynamics^45^, with only a marginal increase in computational cost^46–54^.

While there have been important efforts to automate the procedure for computing Hubbard parameters using LR-cDFT and Hartree-Fock-based methods, to the best of our knowledge no automated workflow exists that allows to determine Hubbard parameters (including the intersite *V*) in a self-consistent fashion. In a recent high-throughput study on binary oxides^55^ the authors implemented a workflow in Atomate^56^ to compute Hubbard *U* and Hund’s *J* parameters using the supercell LR-cDFT approach based on finite differences. MacEnulty and coauthors developed a feature-rich post-processing routine for ABINIT that orchestrates the calculation of Hubbard *U* and Hund’s *J* parameters again relying on LR-cDFT^57^. A different approach is employed by the ACBN0 functional^34^, implemented in Octopus^12,58^ and AFLOW^59,60^, where the self-consistent calculation of *U* and *V* parameters is performed at runtime during the self-consistent field energy minimization. This approach is appealing but uses a different assumption for the first-principles calculation of the Hubbard parameters. Furthermore, the current implementation of intersite interactions^12^ in ACBN0 is less flexible with respect to the coordination environment of central atoms due to the use of user-defined (constant) radial cutoffs, which may represent a blocking and error-prone step in the context of high-throughput applications. Lastly, we also mention the emergence of machine-learning-based techniques for the determination of Hubbard parameters^61–65^. While this approach offers a promising path, training of machine learning models requires extensive datasets of Hubbard parameters generated through well-defined and reproducible calculations. This is crucial, since the effect of Hubbard corrections not only hinges on the numerical values of the Hubbard parameters but also depends on additional factors such as the choice of Hubbard projectors, the base xc functional, and others^40,66–68^.

Hence, a robust, flexible, and reliable framework is needed that automates the submission of thousands of jobs, independently handles common errors and also embraces the FAIR (Findable, Accesible, Interoperable, Reuseable) principles of data management^69^ that ensure a high degree of reproducibility.

In this work, we present aiida-hubbard, a Python package providing an optimized and automated workflow for the structurally self-consistent calculation of Hubbard *U* and *V* parameters using the HP code^44^ of the Quantum ESPRESSO distribution^70–72^, which leverages DFPT^29,30,73^. The package is devised as a plugin for AiiDA^74–76^, a well-established scalable computational infrastructure developed to carry out complex computational workflows while facilitating data provenance. To store the data, we implement HubbardStructureData, a general and flexible data structure in Python that aims at enhancing the reproducibility of Hubbard-corrected DFT calculations. In aiida-hubbard, the execution of workflows can be fully customized by the user; for instance, it can be specified whether or not the self-consistency cycle shall involve a geometry optimization step (including atomic positions, lattice vectors, or both at the same time). We demonstrate the scalability and reliability of the package by computing self-consistently the Hubbard *U* and *V* parameters of 115 structurally diverse Li-bearing crystalline solids composed of up to five different elements. Notably, for the successful workflows only in 6% of the submitted calculations computational errors occurred, all of which were handled successfully without any human intervention. Importantly, our analysis reveals that both the oxidation state (OS) and the coordination environment of the Hubbard atoms independently affect the numerical values of the self-consistent Hubbard *U* and *V* parameters.

For the remainder of this section, we briefly summarize the most essential concepts and notations associated with DFT+*U*+*V*^11,46^ and DFPT^29,30^ which are used throughout this study. Fundamentally, the physical justification for Hubbard *U* corrections lies in their capability to mitigate spurious deviations from the piecewise linearity (PWL) of the DFT total energy with respect to fractional addition or removal of charge^6,27,28,39,77–79^, which are related to electron SIEs^39^. In DFT+*U*+*V*, such deviations from PWL are tackled by adding a penalty term *E*_*U*+*V*_ to the Kohn-Sham (KS) DFT energy^11^:

1 $${E}_{{\rm{DFT}}+U+V}={E}_{{\rm{DFT}}}+{E}_{U+V}.$$

*E*_*U*+*V*_ contains two corrections: (i) an onsite term that penalizes the fractional (i.e., non-idempotent) occupation of orbitals centered on atomic sites, and (ii) an intersite term which stabilizes the occupation of states that are linear combinations of atomic orbitals centered on different (usually neighboring) atoms. It reads:

2 $${E}_{U+V}=\frac{1}{2}\sum _{I}\sum _{\sigma m{m}^{{\prime} }}{U}^{I}\left({\delta }_{m{m}^{{\prime} }}-{n}_{m{m}^{{\prime} }}^{II\sigma }\right){n}_{{m}^{{\prime} }m}^{II\sigma }-\frac{1}{2}\sum _{I}\mathop{\sum }\limits_{J(J\ne I)}^{* }\sum _{\sigma m{m}^{{\prime} }}{V}^{IJ}{n}_{m{m}^{{\prime} }}^{IJ\sigma }{n}_{{m}^{{\prime} }m}^{JI\sigma },$$

where *m* and ${m}^{{\prime} }$ are magnetic quantum numbers associated with the localized manifold being targeted by the correction, *I* and *J* are the atomic site indices, while *U*^*I*^ and *V*^*IJ*^ are the effective onsite and intersite Hubbard parameters, respectively. For the second term of Eq. (2), the sum over *J* is restricted to cover only those neighbors of each atom *I* for which a *V* parameter has been specified (as indicated by the star). For practical calculations, one must define a *Hubbard manifold* to which the *U* and *V* corrections are applied. Traditionally, onsite manifolds comprise entire valence *d* shells of TM elements and/or *f* shells of lanthanides and actinides. Other shells, such as the *p*-shells of chalcogenides and halogenides, may also be targeted^12,67,80^. Moreover, in some works Hubbard corrections have been applied concurrently to multiple shells localized on the same Hubbard atom^11^, or to smaller subsets of the magnetic quantum orbitals of a shell^66,81,82^. We note that Eq. (2) shows the formalism for collinear spin polarization, and refer the reader to Ref. ^48^ for the non-collinear case. The occupations ${n}_{m{m}^{{\prime} }}^{IJ\sigma }$ are obtained by projecting the KS wavefunctions ${\psi }_{v,{\bf{k}}}^{\sigma }({\bf{r}})$ onto localized orbitals ${\phi }_{m}^{I}({\bf{r}})$:

3 $${n}_{m{m}^{{\prime} }}^{IJ\sigma }=\sum _{v,{\bf{k}}}{f}_{v,{\bf{k}}}^{\sigma }\left\langle {\psi }_{v,{\bf{k}}}^{\sigma }| {\phi }_{{m}^{{\prime} }}^{J}\right\rangle \left\langle {\phi }_{m}^{I}| {\psi }_{v,{\bf{k}}}^{\sigma }\right\rangle ,$$

where *v* and *σ* are the band and spin labels of the KS states, respectively, **k** indicates points in the first Brillouin zone (BZ), ${f}_{v,{\bf{k}}}^{\sigma }$ are the occupations of the KS wavefunctions, and ${\phi }_{m}^{I}({\bf{r}})\equiv {\phi }_{m}({\bf{r}}-{{\bf{R}}}_{I})$ are localized orbitals centered on the *I*^th^ atom at the position **R**_*I*_. It is important to recall that the choice of the projector functions exerts a strong influence on the numerical values of calculated Hubbard parameters and affects the prediction of materials properties^67,68,83^. Besides the localized atomic orbitals *ϕ* appearing in Eq. (3), there are other types of projector functions that may provide a more system-specific description of orbital occupations at some expense of computational and conceptual simplicity. Particularly noteworthy in this context are Löwdin-orthogonalized atomic orbitals^83,84^ as well as Wannier functions^66,85,86^ (e.g., maximally localized ones^87–91^). A more detailed discussion of these projector functions including specific advantages and drawbacks can be found in Ref. ^83^.

To carry out practical calculations using the energy functional of Eq. (2), the Hubbard parameters *U* and *V* must be determined for all of the selected target manifolds. The DFPT approach employed in this work evaluates these parameters based on the heuristic finding (demonstrated so far only for the onsite *U*) that Hubbard corrections can (*locally*) eliminate the spurious deviations of the total energy from PWL^28,79^:

4 $${U}^{II}={\left.\frac{{\partial }^{2}{E}_{{\rm{DFT}}}}{\partial {[{\rm{Tr}}({{\bf{n}}}^{II})]}^{2}}\right|}_{q},\,{V}^{IJ}={\left.\frac{{\partial }^{2}{E}_{{\rm{DFT}}}}{\partial {[{\rm{Tr}}({{\bf{n}}}^{IJ})]}^{2}}\right|}_{q},$$

where ∣_*q*_ means that the expressions shall be evaluated at a fixed total charge *q* of the system and ${\rm{Tr}}({{\bf{n}}}^{IJ})$ is the trace of the occupation matrix **n**^*IJ*^ (where **n**^*IJ*^ = ∑_*σ*_**n**^*IJσ*^, and **n**^*IJσ*^ is the matrix whose elements are ${n}_{m{m}^{{\prime} }}^{IJ\sigma }$), whose elements are obtained from Eq. (3). Because a direct control of orbital occupations is not always tractable, instead of computing the response of *E*_DFT_ to changes in the occupation matrix, one computes the response of the occupation matrices to a linear perturbation *α*^*J*^^28^,

5 $${\chi }^{IJ}=\frac{\partial [{\rm{Tr}}({{\bf{n}}}^{II})]}{\partial {\alpha }^{J}},$$

where *χ* is the self-consistent response matrix. This is obtained at self-consistency of the DFPT calculation^29^. In Refs. ^92,28^, it was noted that by varying the site occupations through perturbations, the localized orbitals rehybridize (at least in SCF codes where the interacting part of the potential is updated in every step), giving rise to a change in the energy of the system that is unrelated to deviations from PWL. This *non-interacting*, *bare* curvature, i.e., the response before the self-consistent readjustments of the Hartree and xc potentials due to the perturbation, is also computed as

6 $${\chi }_{0}^{IJ}=\frac{\partial \left[{\rm{Tr}}{({{\bf{n}}}^{II})}_{0}\right]}{\partial {\alpha }^{J}}$$

where *χ*_0_ is the bare response matrix. We note that in order to derive Hubbard *V*^*IJ*^ parameters consistent with Eq. (4), in Eq. (5) one should use the responses of ${\rm{Tr}}[{{\bf{n}}}^{IJ}]$ instead of ${\rm{Tr}}[{{\bf{n}}}^{II}]$. However, the current Quantum ESPRESSO implementation of DFPT relies on Eq. (5) and we expect this inconsistency to have a negligible influence on the numerical values of the resulting *V*^*IJ*^ parameters. With the response matrices from Eq. (5), the Hubbard parameters *U* and *V* can be computed according to Refs. ^11,28^:

7 $${U}^{I}={\left({\chi }_{0}^{-1}-{\chi }^{-1}\right)}^{II},\quad {V}^{IJ}={\left({\chi }_{0}^{-1}-{\chi }^{-1}\right)}^{IJ}.$$

We note in passing that the “full” inversion of the *χ* and *χ*_0_ matrices as practiced in Eq. (7) and used throughout this work is not the only way of computing Hubbard parameters, and that other possibilities have been explored^55,93,94^. Furthermore, we do not include the additional row and column in the response matrices that are sometimes introduced to account for the so-called “background” contribution^28^. For manifolds that respond to perturbations very weakly (such as *d*^10^ ions) or that display a strong intra-shell screening^82^, the linear-response approach presented here can result in oscillating or diverging results and must be used with great care^40,95,96^.

Within the DFPT formalism, the linear responses of Eq. (7) can be conveniently expressed in terms of monochromatic perturbations modulated with wave vectors **q** as^29^:

8 $$\frac{\partial {n}_{m{m}^{{\prime} }}^{I\sigma }}{\partial {\alpha }^{J}}\equiv \frac{\partial {n}_{m{m}^{{\prime} }}^{sl,\sigma }}{\partial {\alpha }^{{s}^{{\prime} }{l}^{{\prime} }}}=\frac{1}{{N}_{{\bf{q}}}}\mathop{\sum }\limits_{{\bf{q}}}^{{N}_{{\bf{q}}}}{e}^{i{\bf{q}}\cdot \left({{\bf{R}}}_{l}-{{\bf{R}}}_{{l}^{{\prime} }}\right)}{\Delta }_{{\bf{q}}}^{{s}^{{\prime} }}{n}_{m{m}^{{\prime} }}^{s\sigma },$$

where *s* and ${s}^{{\prime} }$ indicate the atomic indices within the unit cell, while *l* and ${l}^{{\prime} }$ are the unit cell indices, such that *I* ≡ (*sl*) and $J\equiv ({s}^{{\prime} }{l}^{{\prime} })$, *N*_**q**_ is the total number of **q** points, ${\Delta }_{{\bf{q}}}^{{s}^{{\prime} }}{n}_{m{m}^{{\prime} }}^{s\sigma }$ is the lattice-periodic response of occupation matrices to the **q**-specific perturbation, **R**_*l*_ and ${{\bf{R}}}_{{l}^{{\prime} }}$ are the Bravais lattice vectors. Further details can be found in Refs. ^29,30^. This approach allows to avoid using computationally expensive supercells, making it the method of choice for large-scale applications.

## Results

### General structure of the aiida-hubbard plugin

**Fig. 1:**
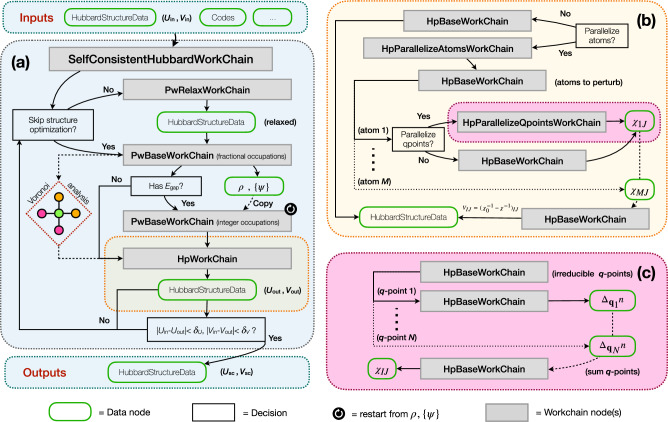
Schematic illustration of the aiida-hubbard plugin that automates the self-consistent calculation of the Hubbard *U* and *V* parameters. **a** The main SelfConsistentHubbardWorkChain workflow which automates the self-consistent calculation of *U* and *V* parameters. It iterates (optionally) structural optimizations via the PwRelaxWorkChain, ground-state calculations via the PwBaseWorkChain, and the DFPT calculations of Hubbard parameters via the HpWorkChain. In particular, the latter can be used to fully exploit the parallel capabilities of the HP code^44^, i.e. by (optionally) first parallelizing the calculations over inequivalent Hubbard atoms to perturb, using the HpParallelAtomsWorkChain (panel (**b**)), and then (optionally) parallelizing over irreducible monochromatic perturbations (**q** points) via the HpParallelQpointsWorkChain (panel (**c**)). These nested calls are visualized by the different colored boxes.

The workflow shown in Fig. 1 contains several key building blocks. The main self-consistent Hubbard workflow and its subprocesses are implemented as AiiDA *workchains*^76^, powering the automated handling and reproducibility of all the calculations. These workchains are represented by grey rectangles. Data nodes, representing inputs and outputs of the workflows and calculations, are depicted by green rounded boxes. For clarity, not the entire nested list of inputs is shown in the figure, but only the mandatory input data classes that are needed to run the workflow. The light blue box (Fig. 1a) contains the outline of the SelfConsistentHubbardWorkChain, the main workflow of the package, which carries out the self-consistent calculation of the Hubbard parameters. Its “child” processes are the PwBaseWorkChain and the PwRelaxWorkChain, which are specialized workchains that run the PW code (pw.x executable)^70^ of Quantum ESPRESSO as part of the aiida-quantumespresso plugin^97^, and the HpWorkChain managing the parallel capabilities of the HP code (hp.x executable)^44^ of Quantum ESPRESSO. The orange and pink boxes (Fig. 1b and c) zoom in on the fine-grained parallelization facilitated by the DFPT framework and the HP code. The main input and output of the workflow is a HubbardStructureData object, a new data type created to store information on the Hubbard functional together with the atomistic structure.

### Joint description of atomistic structures and Hubbard interactions

To fully describe the Hubbard part of the inputs to a DFT+*U*+*V* calculation (such that the latter becomes reproducible), it is not sufficient to merely store the numerical values of the interaction parameters. First and foremost, several prescriptions of Hubbard corrective functionals (sometimes referred to as *flavors*) have been developed over the years, the most prominent ones being the rotationally invariant formulation by Dudarev et al.^10^ and the approach presented by Liechtenstein and coworkers^8^. It is intuitive that the use of different corrective terms can lead to different results. Another critical component is the choice of Hubbard projectors (e.g., nonorthogonalized atomic orbitals, orthogonalized atomic orbitals, Wannier functions, etc.), which again has a decisive impact on the results of a DFT+*U*+*V* calculation. Finally, every set of Hubbard parameters used in a calculation is tied to the atomistic structure to which it is applied. Therefore, HubbardStructureData unifies the description of Hubbard corrections and the respective atomistic structures in one class, and provides user-friendly auxiliary utilities that facilitate the initialization and handling of Hubbard-related data. This is achieved by combining the structural information, inherited from the StructureData class already available in AiiDA, with a new Hubbard class presented below.

In Hubbard, we distinguish three key components: the mathematical formulation of the correction (flavor), the Hubbard projectors, and the interaction parameters. The Hubbard formulation (e.g., “Dudarev”^10^ or “Liechtenstein”^8^) and the kind of projectors (e.g., “atomic”, “ortho-atomic”^83^) are specified as strings, whereas the interaction parameters are stored as a list of instances of HubbardParameters. We decide not to include the pseudopotentials in Hubbard since, thanks to the provenance model of AiiDA, this piece of information can be easily traced back or simply added as extra metadata, thus avoiding inefficient repetition of data. Nevertheless, we recall the impact of pseudopotential choice (to be more precise, the atomic orbital projectors stored in the pseudopotential file) on the numerical value of Hubbard parameters computed using LR-cDFT, which can vary by 2 − 3 eV^40^.

**Fig. 2:**
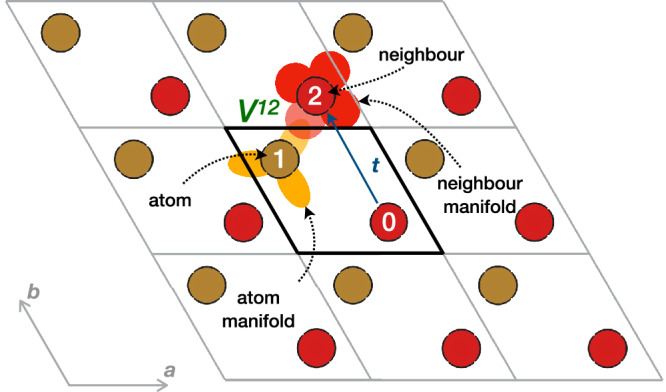
Two-dimensional periodic system summarizing the quantities needed to describe a Hubbard interaction *V*^*IJ*^. This example shows a 2D lattice with two crystallographically inequivalent atoms in the unit cell, indexed 0 and 1. To store a Hubbard interaction between atoms 1 and 2, where 2 is a periodic image of atom 0, one stores the indices 1 and 0 along with the translation vector **t** that maps atom 0 onto atom 2 using integer multiples of the cell vectors **a** and **b** (in this case, **t** = (0, 1)). Moreover, the yellow and red orbitals shown around atoms 1 and 2 indicate the target Hubbard manifolds, which must also be stored. Finally, *V*^12^ represents the value of the interaction parameter in energy units, usually expressed in eV.

HubbardParameters is an extra class defining a single Hubbard interaction that contains its type (*U*, *V*, *J* etc.), the indices and manifolds (e.g. 3*d*, 2*p*, etc.) of the atom(s) involved, as well as the value of the respective parameter expressed in energy units (eV). To allow for the description of interactions between two distinct atoms (intersites), HubbardParameters additionally stores a second atomic index and a second target manifold. For calculations with periodic boundary conditions, intersite couples might be located in periodic images of the unit cell. Therefore, the first atomic index is always referenced within the unit cell, while every second atomic index is augmented by a translation vector **t** pointing to the atom’s corresponding periodic image. For instance, for the structure shown in Fig. 2, an intersite interaction between atoms 1 and 2 (*V*^12^) would be stored as an interaction between atoms 1 and 0 plus the translation vector that maps atom 0 onto atom 2. The *U* and any other onsite interaction parameters can be defined by specifying the same index and manifold for both fields, and by assigning a null translation vector **t** = **0**.

### Choosing the interacting Hubbard couples

Before intersite Hubbard *V*^*IJ*^ parameters can be evaluated and applied, it is necessary to define the interaction couples by providing their atomic indices and target manifolds. Since intersite corrections are intended for systems where orbital hybridization plays an important role^11^, *V*^*IJ*^ parameters are typically established for nearest-neighbor couples (e.g., between the *d*-shell of a central TM atom and the *p*-shells of its ligands). In practice, it is desirable to let the user specify the elements to be considered as interacting, while delegating the (potentially error-prone) determination of the respective atomic indices *I* and *J* to an algorithm. However, generally the search for nearest neighbors can be challenging, particularly in structures hosting simultaneously different coordination environments (e.g., tetrahedral and octahedral sites in spinels). Counting the neighbors contained in a sphere around each central atoms offers a straightforward solution but necessitates a common radial cutoff value that must be at the same time large enough to include all of the specified couples but small enough not to introduce additional interactions. Such a common cutoff radius can be hard to determine, or might not even exist at all; for instance in amorphous and low-symmetry ordered structures, where coordination environments are notoriously difficult to characterize. An additional problem occurs in workflows involving structural optimizations: when the cutoff is recalculated following a structural relaxation, it cannot be guaranteed that the same (and only the same) atoms are contained in the new sphere. These issues become particularly unmanageable in high-throughput calculations, thus motivating the need for a robust automation of the process, which should be carried out in each iteration of the self-consistent cycle. In aiida-hubbard, the analysis of nearest-neighbours is therefore performed using the Voronoi tessellation method^98^ as implemented in the Pymatgen core utilities^99,100^. This parameter-free approach systematically accounts for diverse coordination environments, even if these coexist in the same atomistic structure, without the need for a common radial cutoff.

### Description of the SelfConsistentHubbardWorkChain

Having established a consistent data structure for storing Hubbard data and having automated the determination of intersite Hubbard couples, we now present the core workflow of aiida-hubbard. The SelfConsistentHubbardWorkChain combines the capabilities of the PwBaseWorkChain and PwRelaxWorkChain^75,97^ with the HpWorkChain. The self-consistency of the Hubbard parameters^30,42^ is achieved iteratively by performing (i) structural optimizations, (ii) single-point DFT+*U*+*V*, and (iii) DFPT calculations of *U* and *V* until convergence. After each structural optimization, the relaxed structure is used to perform a single-point DFT+*U*+*V* calculation with fractional electronic occupations (indicated by “fractional occupations” in Fig. 1a) in order to identify whether the system is metallic or insulating. If the electronic structure displays a finite band gap, an extra calculation with fixed integer occupations is performed (indicated by “integer occupations” in Fig. 1a), which reuses the previously obtained charge density and wavefunctions in order to accelerate convergence and to preserve the determined magnetic ground state in case of spin-polarized calculations. This second single-point step is fundamental to avoid numerical divergence in the DFPT calculation at **q** = **0**^44,73^. Finally, the DFT+*U*+*V* ground-state is used to carry out the DFPT calculation that predicts the new set of Hubbard parameters. After completing a cycle, these Hubbard parameters are then used for the next iteration. This sequential procedure is repeated until the variations in parameters fall below user-predefined thresholds *δU* and *δV* (typically in the range of 0.01 to 0.1 eV).

We note that other ways of conducting the self-consistency procedure are also possible. For instance, the structural optimization can be omitted so that the Hubbard parameters are converged or at least iterated a couple of times for a fixed atomistic structure. Alternatively, an intermediate strategy could be pursued in which structural optimizations are not performed at every cycle, but instead intermittently (e.g., only once every 3-5 iterations). Another potentially useful approach that might reduce the number of iterations involves using a reasonable guess for the Hubbard parameters instead of starting from the initial values *U*_in_ = *V*_in_ = 0. Initial values can either stem from a machine-learning model^64^ or can be chosen empirically. For heavily oscillating Hubbard parameters, a mixing strategy can be introduced. The origin of the oscillations can be attributed in part to the *dU*/*d***R** contribution (usually discarded) to the forces, which can have a sizeable effect on structural relaxation^101^.

### Parallelization levels of the HpWorkChain

A crucial aspect for practical applications of the SelfConsistentHubbardWorkChain is the computational time required to complete an iteration. As the most demanding step of each cycle generally consists in the computation of the Hubbard parameters with DFPT, finding strategies to accelerate the latter is desirable. While the HP code of Quantum ESPRESSO provides several options that allow for a distribution of the computational load, some of these parallelization levels can be coordinated by a high-level orchestrator. For this purpose, aiida-hubbard implements the HpWorkChain, which allows the user to parallelize the DFPT calculations in an automated fashion using up to two levels of parallelization. The first layer, a parallelization over atoms, arises because each element *IJ* of the response matrix *χ* can be computed independently from the others (see Eq. (7)). This functionality is provided by the HpParallelizeAtomsWorkChain (see Fig. 1b). The second level of parallelization can be achieved using the HpParallelizeQpointsWorkChain (Fig. 1c), which distributes the calculation of the independent wavevectors **q** that contribute to the total occupation matrix (see Eq. (8)). Particularly for large systems, leveraging both strategies can offer a computational boost on massively parallel architectures, where the DFPT calculations for each perturbed Hubbard atom and **q**-point can be executed concurrently on the available compute nodes. The independent DFPT calculations spawned by the parallel workchains are managed by the HpBaseWorkChain, a “base” workflow designed to run the hp.x binary of the HP code featuring automated submission, retrieval and error handling capabilities. Errors are addressed effectively by modifying the inputs without any user intervention, which is crucial for high-throughput calculations. For example, if the self-consistent response does not converge within the maximum number of iterations, HpBaseWorkChain submits a new *hp.x* job with a lower mixing factor for the response charge density mixing needed for solving iteratively the Sternheimer equations of DFPT^29,30^.

### Semi-automatic input preparation

In the preceding sections, we have conceptualized workflows to perform self-consistent calculations of Hubbard parameters. However, the results of both the workflows and the individual DFT+*U*+*V* and DFPT calculations depend upon a large number of inputs. These inputs comprise code-specific keywords such as convergence thresholds (on energy, forces, stresses), cutoff values, mixing parameters, **k**-point grids for the Brillouin zone sampling, pseudopotentials, but also metadata associated with the computational resources including the walltime limit and the number of computational nodes and cores requested, to name a few. Not only for non-expert users, choosing suitable values for all of the inputs and generating (syntactically correct) input files can be a tedious and error-prone task. To reduce this complexity, aiida-hubbard features a get_builder_from_protocol method for each of the workchains^97,102^. This method automatically populates the inputs, while the user is left with the task of providing only three indispensable pieces of information: (i) an instance of HubbardStructureData (i.e. the atomistic structure with initialized Hubbard parameters), (ii) AiiDA code instances containing information on how to run the PW and HP codes^75,76^, and (iii) a string defining in a general fashion the accuracy of the calculation called protocol (“fast”, “balanced”, or “stringent”)^103^. A summary of the main calculation parameters these protocols *initialize* is reported in Supplementary Table 1. Importantly, after calling the get_builder_from_protocol method, the user receives a pre-populated set of inputs, which can then be checked and modified before being used for the execution of the workflow.

### Impact of structural optimizations on self-consistent Hubbard parameters

In the outline of the SelfConsistentHubbardWorkChain, we have presented different strategies for the self-consistent computation of Hubbard parameters. Hence, it is worthwhile to investigate the impact of different schemes on the numerical value of the Hubbard parameters. Here, we perform numerical experiments in which we compare two commonly employed approaches: (*i*) converging the Hubbard parameters by alternating single-point DFT+*U*+*V* and DFPT calculations, and (*ii*) by performing the optimization of the lattice parameters and the atomic positions at the beginning of each cycle. In the following we will refer to strategy (i) as *NR scheme* (no relaxation) and (ii) as *FR scheme* (full relaxation). For the sake of simplicity, we conduct these experiments by computing only the self-consistent Hubbard *U* parameters, neglecting intersite *V* interactions. For the FR scheme, we omit the structural optimization on the first iteration, though we emphasize that this is optional in the workflow settings. We apply these two schemes to six chemically and structurally diverse crystalline solids containing Li and Fe that have been investigated experimentally^104^. We apply onsite *U* corrections to the Fe-3*d* states, and converge the *U* parameter within *δU* = 0.1 eV.

**Fig. 3:**
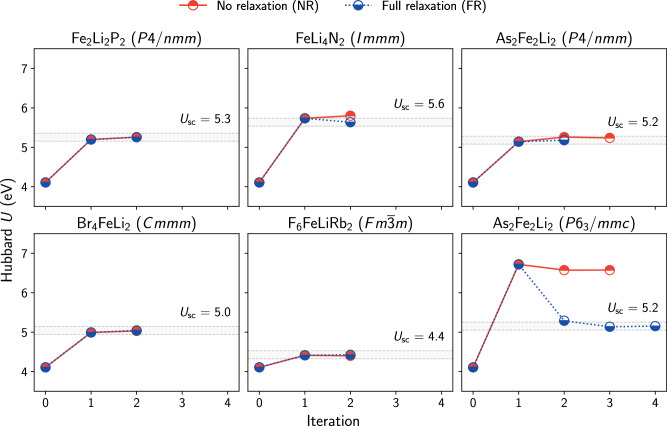
Self-consistent convergence of Hubbard *U* for Fe-3*d* using two different schemes. Values of Hubbard *U* for six different bulk Fe-containing solids as a function of the iteration of the self-consistent cycle, using the NR (red) and FR (blue) schemes. For each compound, the associated Hill formula and space group are reported in the title of the corresponding panel. Iteration 0 corresponds to the starting guess *U*_Fe_ = 4 eV. Geometry optimizations of the FR cycle were omitted in iteration 1. The gray shaded area shows the convergence range *U*_sc_ ± *δU* (*δU* = 0.1 eV), around the final values of the FR cycles.

Figure 3 shows the computed numerical values of *U* during the self-consistency cycle, with red and blue half-filled dots referring to the NR and FR schemes, respectively. The data points at iteration 0 represent the starting guess *U*_Fe_ = 4 eV that was used to initialize the self-consistent cycles. We find that the numerical values of *U* computed at iteration 1 are always identical for both approaches since the geometry optimizations of the FR scheme were omitted at this iteration. It can be observed that the final self-consistent values of *U*, reported as *U*_sc_, depend on the specific compound and range from 4.4 to 5.6 eV. Interestingly, none of the two approaches consistently outperforms the other with respect to the number of iterations required to converge *U*. Furthermore, the two strategies yield the same *U*_sc_ values for all but one structure (As_2_Fe_2_Li_2_ with the space group *P*6_3_/*mmc*), where the *U*_sc_ parameter obtained for the NR approach exceeds that of the FR approach by about 1.4 eV. This observation can be explained with a peculiar volume expansion by over 150% upon optimization of the crystal structure, which concurs with significant changes in the electronic structure. In fact, the total projected occupation of the Fe-3*d* shell decreases by about 1*e*^−^ following the volume expansion. Since the Hubbard parameters are calculated from the response of the occupation matrices, the change of the latter leads to a shift in the computed *U* value. The large increase in volume results from the initial experimental structure being measured under high-pressure conditions (*P* > 1000 kPa)^104^. All of the other structures display less pronounced volume deviations (14% maximum and 6% on average), and also present only negligible changes in the occupations of the Fe-3*d* manifold. Thus, in these cases, the FR and NR strategies yield the same *U*_sc_ parameters (within the chosen threshold). A similar observation can be made in an analogous numerical experiment carried out with Mn-bearing compounds, whose results are presented in Supplementary Fig. 1.

Therefore, the impact of structural optimization on self-consistent Hubbard parameters is negligible for most of the materials considered here. However, this should not be assumed as a general trend, as the importance of the geometry optimization depends on the magnitude of rearrangements between the final and starting atomistic structures.

### Trends in Hubbard parameters from 105 Li-bearing materials

Having demonstrated the flexibility of the workflows to account for different self-consistent strategies, we now proceed to showcase their scalability and robustness by carrying out calculations of Hubbard *U*_sc_ and *V*_sc_ parameters across a diverse set of materials. While several mid- and high-throughput studies on the prediction of Hubbard parameters can be found in literature^55,67,80,85,105^, these were limited to the prediction of *U* (and sometimes *J*) parameters only, not to mention computational difficulties encountered due to the supercell approach required by LR-cDFT in some of these studies. The present study focuses on 115 experimentally known crystalline solids containing Li, Fe or Mn, and not limiting additional elements, with unit cells of 32 atoms or fewer. The full list of materials studied is presented in Supplementary Table 2. These compounds are relevant as they represent potential candidates for cathodes in novel Li-ion batteries. Moreover, it has been shown that the DFT+*U*+*V* approach can accurately predict various properties of such materials, including open-circuit voltages^42,43,51^.

We compute the onsite *U* parameters for the TM 3*d* shells, and consider intersite *V* interactions between the TM 3*d* shells and the *p* shells of neighboring chalcogenide atoms (O, S, Se, Te). This choice is based on the expectation that inter-atomic orbital hybridization (e.g., the formation of *σ*_*p*−*d*_ states) is most pronounced for these couples. Due to the absence of chalcogenides in 42 of the 115 structures, only the onsite *U* parameter is computed for these cases. To not only obtain the self-consistent Hubbard parameters but also the structural DFT+*U*(+*V*) ground states, we leverage the FR scheme presented in the previous section, initializing the workflows with *U*_TM_ = 5.0 eV and *V* = 0 eV, when applicable. We keep omitting the first structural optimization when employing the FR scheme to obtain the Hubbard parameters at the experimental structural configuration. Furthermore, Fig. 3 (and Supplementary Fig. 1) suggests that the first calculation of the Hubbard parameters gives results close to the self-consistent numerical values, thus allowing one geometry optimization step to be saved. All but ten of the submitted SelfConsistentHubbardWorkChain processes finished successfully, managing the automated recovery of several computational errors that occurred in about 6% of the DFT+*U*+*V* and DFPT calculations submitted. The self-consistent cycles converged within 2.9 iterations on average. Thus, about three structural optimizations, single-point DFT+*U*+*V* calculations, and DFPT runs are needed to converge the Hubbard parameters within the *δU* = 0.1 and *δV* = 0.1 eV thresholds. In more detail, 34 workchains converged in 2 iterations, 58 workchains needed 3 iterations, and only 13 required 4 or more iterations to reach self-consistency. Among the unsuccessful calculations using the workflow, many failed due to crashes of the PW and/or HP simulations caused by non-trivial numerical issues (e.g., with the minimization algorithms). While future updates to the Quantum ESPRESSO distribution or adjustments in aiida-quantumespresso and aiida-hubbard may address these issues, we identified two compounds where convergence of the Hubbard parameters could not be achieved for physical reasons. We examine these cases in more detail in the Supplementary Discussion and Supplementary Table 3.

**Fig. 4:**
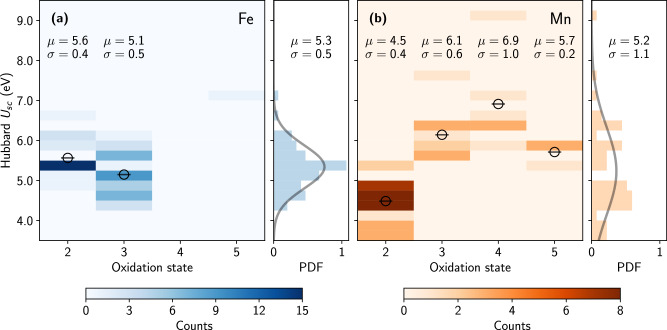
Distributions of self-consistent Hubbard *U*_sc_ parameters among 105 Li-bearing materials. **a, b** Values of onsite Hubbard *U*_sc_ parameters as a function of the OS of Fe and Mn. The side panels show the data probability distribution along with the fitted Gaussian distributions (gray lines) of *U*_sc_ across the explored oxidation states and report the mean values (*μ*) and standard deviations (*σ*) in units of eV.

Figure 4 shows the range of self-consistent Hubbard parameters determined by the workchains, clearly indicating a dependence of *U*_sc_ on the OS of the TM elements. The OSs are determined following the approach of Ref. ^106^ using a threshold of 0.8, i.e., only those eigenstates of the occupation matrix **n**^*IIσ*^ whose eigenvalues *λ*^*σ*^ are determined to be larger or equal to 0.8 are counted as occupied orbitals that determine the OS. Since Fe and Mn are multivalent elements, the OSs they exhibit vary depending on the specific compound. In the vast majority of cases Fe is present as Fe^2+^ or Fe^3+^, whereas the rarer Fe^5+^ species was identified only for one material in our dataset, namely FeLa_2_LiO_6_ (c.f. ICSD entry 252554 and MaterialsCloud ID mc3d-47750/pbe-v1). Conversely, Mn displays more variety. Compounds were found for all OSs between + 2 and + 5 ( + 7 in one material, not shown in the figure; see Supplementary Table 2), with the majority of cases corresponding to Mn^2+^. Interestingly, the mean values of the onsite *U*_sc_ parameters for Fe and Mn are close between each other, respectively 5.3 and 5.2 eV, and are within the typical range of empirical Hubbard *U* parameters used in the literature^107–109^. However, Fig. 4 clearly illustrates that a universally transferable Hubbard parameter does not exist, not even when identical Hubbard projector functions are used, as in this study. In fact, it can be seen that *U* significantly depends on the OS of the TM ion. For instance, the average value of *U*_sc_ for Mn^2+^ is 4.5 eV, whereas that of Mn^4+^ amounts to 6.9 eV. This observation is further supported by the fact that the data distributions do not show a symmetric Gaussian shape of the *U* values, especially when compared to the fitted Gaussian probability distribution functions (reported as gray lines), and instead indicate a clustering due to the distinct OSs, especially for Mn. Nevertheless, the wide range of *U* values found even for compounds with identical OSs suggests that there must be other factors playing an important role.

**Fig. 5:**
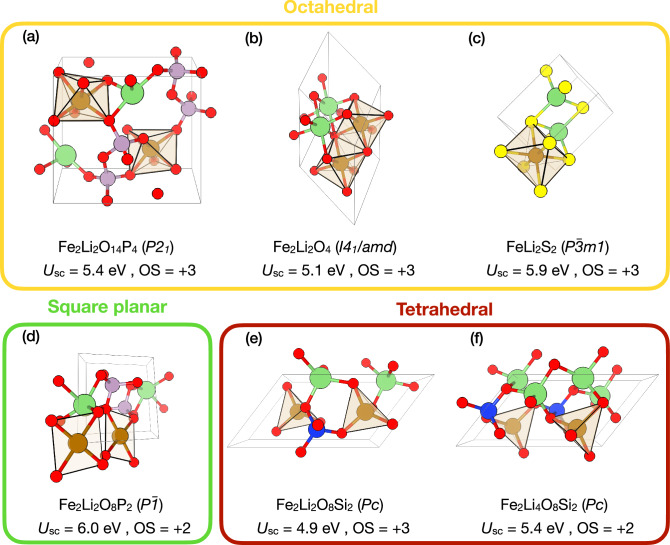
A selection of structurally and chemically diverse compounds containing both Fe and Li. **a-f** Images of the unit cells along with the corresponding Hill formulae, space group symbols in parenthesis, the final self-consistent Hubbard parameters *U*_sc_, and the OSs of the Fe ions. The images were rendered using VESTA^119^. Color code: Fe (brown), O (red), Li (green), Si (blue), P (purple).

We therefore inspect a few representative structures in more detail and present them in Fig. 5. In particular, we focus on six ferric or ferrous compounds that vary not only with respect to the OS ( + 2 or + 3) but also display chemically and structurally distinct coordination environments around the Fe central atom. Analyzing Fig. 5, one can distinguish the following cases:

- *Different coordination environment, same OS, same ligand species*: The Fe atoms in couples (a) and (e) as well as (d) and (f) display the same OSs and their ligand fields are composed of oxygen atoms, however, their coordination geometries differ. This alone leads to significant variations in *U*. For instance, *U* = 5.4 eV for the tetrahedrally coordinated Fe in Fe_2_Li_4_O_8_Si_2_, whereas *U* amounts to 6.0 eV in Fe_2_Li_2_O_8_P_2_, where Fe exhibits a square planar coordination geometry. Hence, here we observe the variation in *U* of 0.5–0.6 eV.
- *Same coordination environment, same OS, different ligand species*: In all of (a), (b) and (c), the Fe atoms are octahedrally coordinated Fe^3+^ species. However, while in (a) and (b) the ligands are O atoms, (c) features S ligands. This again leads to a pronounced increase in *U* by 0.5–0.8 eV when comparing oxides to sulfides.
- *Same coordination environment, different OS, same ligand species*: Compounds (e) and (f) are structurally identical except that (f) contains two additional Li atoms which reduce the OS of (f) from 3 + to 2 +. A difference in *U* of 0.5 eV is consistent with the previously discussed data shown in Fig. 4 and earlier first-principles studies^42,43,51^.
- *Same coordination environment, same OS, same ligand species*: Finally, in spite of the fact that (a) and (b) both posses octahedrally coordinated Fe^3+^ ions, their *U* values still differ by 0.3 eV. Generally, these relatively moderate variations can be attributed to local distortions from the perfect *O*_*h*_ point symmetry, which can be induced by Jahn-Teller effects and other kinds of distortion modes. However, since Fe^3+^ is not Jahn-Teller active, these variations in *U* can be attributed to variations in the volume of the FeO_6_ octahedra induced by the surrounding atoms in the crystal structure and overall changes in the electronic screening of the Fe-3*d* states by those atoms.

This simple and intuitive analysis highlights the strong dependence of *U* on the local environment (in both structural and chemical terms) as well as on the OS of the central atom. In the case of Fe-bearing compounds, these effects lead to variation in *U* less than 1 eV. However, in other compounds like those containing Mn, these variations can have a larger magnitude (see Fig. 4).

**Fig. 6:**
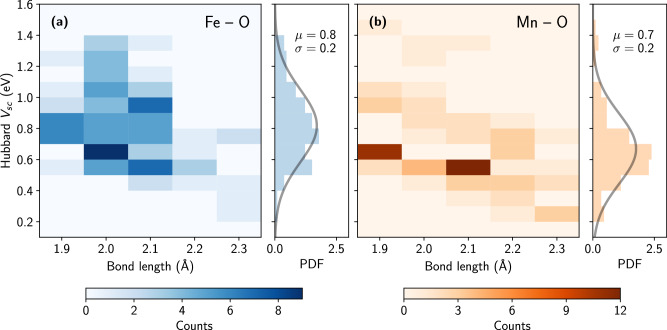
Distributions of self-consistent Hubbard *V*_sc_ parameters among 105 Li-bearing materials. **a, b** Dependence of intersite Hubbard *V*_sc_ parameters computed for the Fe 3*d*-O 2*p* and Mn 3*d*-O 2*p* interactions on the bond length between atoms. The side panels show the probability distribution functions (PDF) along with the fitted Gaussian distributions (gray lines) of *V*_sc_ across the explored bond lengths and report the mean values (*μ*) and standard deviations (*σ*) in units of eV.

Given the clear connection between the onsite *U* parameters and the local environments of the Hubbard atoms, it is intriguing to explore whether the intersite *V*_sc_ parameters display similar dependencies. Since *V*_sc_ parameters are computed for atom pairs, their values might intuitively vary with the inter-atomic distance (bond length) between the interacting partners. Figure 6 shows the distribution of Hubbard *V*_sc_ parameters as a function of inter-atomic distance for Fe-O (panel (a)) and Mn-O (panel (b)) pairs, with corresponding PDFs displayed in the side panels. Other couples were excluded from this analysis as the majority of compounds in the database are oxides (i.e., 60 out of 70 structures containing chalcogenide atoms). However, a list of all compounds and their corresponding Hubbard parameters, including *V*_sc_ for couples with chalcogenides other than O, can be found in Supplementary Table 2. Analyzing Fig. 6, it can be seen that the distributions of *V*_sc_ parameters among the Fe-O (a) and Mn-O (b) couples are quite similar. In general, *V*_sc_ decreases with increasing bond lengths and vice versa. Notably, the *V*_sc_ parameters in Fig. 6 can be described by Gaussian distributions with mean values of 0.8 eV (Fe-O) and 0.7 eV (Mn-O), and a standard deviation of 0.2 eV for both couples. Nonetheless, there are significant variations in *V*_sc_ that cannot be explained by a bond length dependence alone. For example, *V*_sc_ values computed for Fe-O interactions at bond lengths of about 2.0 Å vary between 0.5 and 1.4 eV. Moreover, in Fig. 6b one might distinguish at least two clusters: one that starts at *V* ≈ 0.3 eV for bond lengths of 2.3 Å and ends at *V* ≈ 0.6 eV for distances of 1.9 Å, and a second one ranging from *V* ≈ 0.6 eV (at 2.3 Å) to *V* ≈ 1 eV (at 1.9 Å). As for the onsite *U*, such substantial variations reflect the non-trivial dependence of *V* parameters on both electronic and geometric degrees of freedom.

## Discussion

We have presented aiida-hubbard, a computational framework based on the AiiDA infrastructure^74^ that automates, in a reproducible yet flexible fashion, the self-consistent calculation of onsite Hubbard *U* and intersite Hubbard *V* parameters leveraging the parallel capabilities of DFPT^44^. We devised HubbardStructureData, a FAIR^69^ code-agnostic data type that jointly encodes all information relevant to the Hubbard corrections and the atomistic structure. The difficulty of automatically defining intersite *V* interactions for a generic coordination environment has been solved by exploiting a Voronoi tessellation algorithm at runtime. To showcase the workflow’s capabilities, we employed it to compute Hubbard parameters for 115 Li-bearing bulk solids with potential relevance for electrochemical applications, including Li-ion batteries. As evidenced by the success rate of 91% (105 in 115 workchains), aiida-hubbard is robust and highly reliable due to its integrated and automatic error handling, especially when considering that the selected compounds were quite realistic featuring unit cells of up to 32 atoms, diverse chemistry and bonding environments. An analysis of trends in the distribution of self-consistent Hubbard *U* parameters revealed a strong dependence of the *U* values of 3*d* manifolds on the oxidation states of the respective TM atoms. At the same time, the coordination environment (i.e., number, arrangement and kind of ligands) also exerts substantial influence on *U*. The Hubbard *U* parameter of Fe 3*d* manifolds varies by up to ~ 3 eV, whereas for the more polyvalent Mn larger variations in *U* by ~ 6 eV are observed. In particular, we found characteristic shifts of the numerical value of *U* upon change in oxidation state or coordination environment, respectively, by about 0.5 eV and 1.0 eV for Fe and Mn. Variations in the intersite *V* parameters were smaller, ranging between 0.2 eV and 1.6 eV when considering TM–oxygen pairs. These values correlate with the bond lengths between the interacting atoms, generally decreasing in magnitude as the interatomic distance increases; however, significant fluctuations remain that could not be explained based on distance arguments alone. These observations indicate that the numerical values of *U* and *V* are subject to a complex interplay between electronic and structural degrees of freedom, meaning that the parameters cannot be accurately determined based on oxidation state or coordination environment only. Therefore, machine learning models designed for predicting Hubbard parameters will likely need to incorporate descriptors of both kinds (e.g., using the OS^106^ and ACE^110^ methods) to enhance the predictive accuracy. For this purpose, it would also be desirable to investigate and quantify the impact of the input parameters (**k**- and **q**-point meshes, energy cutoffs, to name a few) on the numerical precision of the resulting *U* and *V* values to develop predefined sets of input parameters with a predictable output precision. The present framework includes three such protocols named “fast”, “balanced” and “stringent”, with their parameterization informed by several prior works by the authors. It is generally advisable to include the geometry optimization step to avoid calculating Hubbard parameters for potentially unexpected ground states and to ensure consistency between the Hubbard parameters and the structure. Specifically, while the differences in *U* values between the workflows with and without structural optimization were minimal for most cases, a notable exception was observed for As_2_Fe_2_Li_2_. In this case, the final *U*_sc_ parameter differed by over 1 eV due to a significant volume expansion during structural optimization.

Beyond such procedural details, a more fundamental aspect that is crucial for the use of Hubbard-corrected DFT methods is the choice of the Hubbard manifold. To date and to the best of our knowledge, no unequivocal prescription has been developed that allows for a rational determination of the latter, i.e., to answer the question *where to apply the U (and the V, J etc.)*. The definition of the Hubbard manifold can influence the prediction of material properties^55,67,82^. For example, results may vary depending on whether *U* corrections are applied to ligand *p* shells or whether the 4*f* shell, the 5*d* shell, or both are targeted in lanthanides. In certain cases, the traditional practice of (exclusively) correcting TM *d* shells can result in diverging or oscillating Hubbard parameters, as observed in two of the unsuccessful workchains (see Supplementary Discussion). Therefore, until a well-defined prescription is developed, based, e.g., on correcting self-interaction errors in the most localized representations, automated DFT+*U*(+*V*) workflows – including the present one – will require human choices for the Hubbard manifolds to linearize the DFT functional. Nonetheless, aiida-hubbard can facilitate the exploration of various Hubbard manifolds in a fast and reproducible way, helping to address this challenge in the future.

Finally, we expect our work to be particularly useful for modeling large systems (e.g., with defects) or for calculating observables whose evaluation potentially requires numerous independent calculations, such as vibrational properties. Both types of applications benefit from the enhanced electronic structure description provided by DFT+*U*(+*V*), which incurs minimal additional computational cost compared to (semi)local functionals. This prospect is supported by the extendable and modular nature of the package, and even more so by the constantly growing universe of AiiDA plugins such as aiida-vibroscopy^102^ and aiida-defects^111^.

## Methods

### Self-consistent calculation of *U* and *V*

All calculations are performed using the aiida-hubbard plugin v.0.1.0 that is run using Quantum ESPRESSO distribution v7.2^44,70–72^. We use the PBEsol flavor^112^ for the spin-polarized GGA xc functional, and employ pseudopotentials from the SSSP library v1.3 (efficiency)^113^. To construct the Hubbard projector functions we use atomic orbitals which are orthogonalized using the Löwdin’s method^83,114,115^. The Brillouin zone is sampled using uniform *Γ*-centered **k**-point meshes with *Δ***k** = 0.2 Å^−1^. The kinetic-energy cutoffs for the expansion of KS wavefunctions are set to the recommended values of the SSSP library^113,116^. The crystal structures are optimized using the Broyden-Fletcher-Goldfarb-Shanno (BFGS) algorithm, with a convergence threshold for the total energy of 10^−6^ Ry/atom, for forces of 10^−4^ Ry/Bohr, and for pressure of 0.5 kbar. For the DFT step prior to each DFPT calculation, we use Marzari-Vanderbilt cold smearing^88^ with a broadening parameter of 0.01 Ry and *Δ***k** = 0.4 Å^−1^. The DFPT calculations of Hubbard parameters are performed using **q**-point meshes with a maximum distance of *Δ***q** = 0.8 Å^−1^. As described above, structural optimizations, single-point DFT+*U*+*V* calculations, and DFPT steps are iterated until self-consistency, which is achieved when the variation of Hubbard parameters fells below *δU* = *δV* = 0.1 eV. At each iteration, only intersite parameters of the full *V*^*IJ*^ belonging to the nearest neighbours of the TM ions are kept for each next iteration. Nearest neighbour analysis is performed using the CrystalNN^117^ class as implemented in Pymatgen^99,100^, which exploits a Voronoi algorithm^98^ to determine the number of nearest neighbours.

## Supplementary information

Supplementary Information

## Data Availability

The data used to produce the results of this work are available in the Materials Cloud Archive^118^.

## Code availability

The code is open source and made available on GitHub (https://github.com/aiidateam/aiida-hubbard). It is also distributed as an installable package through the Python Package Index (https://pypi.org/project/aiida-hubbard/). The base code is open to external contributions for improvements through the GitHub pull request system. The full documentation with tutorials can be found at https://aiida-hubbard.readthedocs.io/en/latest/.
