# Supplementary material for: First-principles Hubbard parameters with automated and reproducible workflows
Source: NPJ Comput Mater. 2025 Jun 16;11(1):183. doi: 10.1038/s41524-025-01685-4 (PMC12170337; doi:10.1038/s41524-025-01685-4)
Supplement: Supplementary file 1 — Supplementary Information [file 41524_2025_1685_MOESM1_ESM.pdf]

Supplementary Information for "First-principles Hubbard parameters with automated and reproducible workflows"

Lorenzo Bastonero,[1,*] Cristiano Malica,[1] Eric Macke,[1] Marnik Bercx,[2] Sebastiaan P. Huber,[3] Iurii Timrov,[2] and Nicola Marzari[1,2,3]

[1] *U Bremen Excellence Chair, Bremen Center for Computational Materials Science, and MAPEX Center for Materials and Processes, University of Bremen, D-28359 Bremen, Germany*

[2] *PSI Center for Scientific Computing, Theory, and Data, and National Centre for Computational Design and Discovery of Novel Materials (MARVEL), 5232 Villigen PSI, Switzerland*

[3] *Theory and Simulation of Materials (THEOS), and National Centre for Computational Design and Discovery of Novel Materials (MARVEL), École Polytechnique Fédérale de Lausanne (EPFL), CH-1015 Lausanne, Switzerland*

(Dated: May 15, 2025)

* lbastone@uni-bremen.de

I. SUPPLEMENTARY DISCUSSION

Analysis of the failed work chains

Here we analyze the ten unsuccessful work chains that resulted from the investigation of the 115 Li-containing bulk structures. An overview of the materials for which the work chain failed, along with additional information on the step of the workflow where it stopped and the reason of the failure, is provided in Supplementary Table III. Two main causes of workflow failure were observed: (i) numerical issues, which account for eight of the ten cases, and (ii) physical issues related to the DFPT approach, causing divergence or oscillation of the computed Hubbard parameters in two cases. At least some of the numerical issues can likely be resolved by further improving the determination and adaption of the input parameters so as to converge even the most difficult ground states. Conversely, to understand the problem behind the workflows that did not converge due oscillating or diverging Hubbard U parameters is less intuitive. Specifically, the "problematic" compounds are the cubic Prussian Blue Analogues $Li_2Cu[Fe(CN)_6]$ and $Cs_2Li[Mn(CN)_6]$ (Hill formulae $C_6CuFeLi_2N_6$ and $C_6Cs_2LiMnN_6$, respectively). The occurrence of unphysical Hubbard U values such as the $U \approx 50\,eV$ observed for $Cs_2Li[Mn(CN)_6]$ can be understood from Eqs. (2) and (5): a Hubbard U^I parameters diverges when $\chi^{II} \rightarrow 0$, i.e., when the perturbation α^J does not induce any measurable change in the occupations $\mathbf{n}^{II}$ of the Hubbard manifold. While it has been recognized quite early that this can be an issue when computing Hubbard U parameters of closed shells [1, 2] (e.g., the $3d^{10}$ shells of Zn^{2+} or Cu^{1+}), only recently attention has been brought to cases where U diverges although the d shell is partially occupied [3]. In fact, a vanishing χ is generally expected whenever the frontier states (HOMO and LUMO) contain no relevant contributions from the Hubbard manifold, e.g., from the TM-d shell. This often applies to insulators of the charge-transfer type and also to compounds with TM ions in low-spin configurations [3, 4]. Moreover, the competition between Jahn-Teller distortions and crystal symmetry constraints may "freeze" electronic response, as is the case for the Mn and Fe species in $Cs_2Li[Mn(CN)_6]$ and $Li_2Cu[Fe(CN)_6]$, respectively (see Fig. 1(a)). This can also be observed in the projected density of states (PDOS) reported for $Cs_2Li[Mn(CN)_6]$ in Fig. 1(b), calculated at the experimental geometry using both PBEsol and PBEsol+U with a fixed value of $U = 5$ eV. The application of the onsite Hubbard U has a negligible effect on

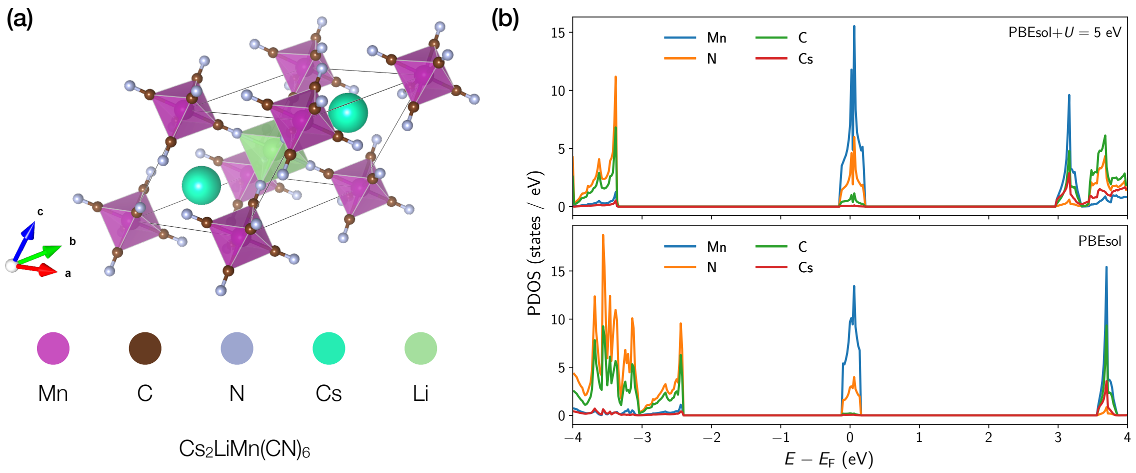

Supplementary Figure 1. (a) Crystal structure and (b) projected density of states (PDOS) of $Cs_2LiMn(CN)_6$ calculated at the initial experimental configuration without structural optimization using PBEsol and PBEsol+U ($U = 5$ eV for the Mn-3d states).

the relative position of the Mn d states in the electronic structure of the material, suggesting the unresponsive behavior of the target Hubbard manifold leading to large numerical values of the Hubbard U parameter from DFPT. Therefore, the failure of these two work chains has physical reasons rather than computational ones, and represents an irrecoverable error. To resolve it, a revised and more suitable definition of the Hubbard manifold is required [3].

Handling of failed calculations in DFPT-based Hubbard workflows

Occasionally, the automated calculation of Hubbard parameters based on DFPT may fail. As reported in Supplementary Table III, some failures encountered in this work occurred already at the DFT+U+V ground state or structural optimization step (i.e. prior to entering the DFPT stage), while some other failures occurred at the DFPT step. While an automated fallback to a supercell-based linear-response approach [5] could, in principle, offer an alternative path, such functionality is not currently implemented in `aiida-hubbard`. This is due to the significant complexity and development effort required, which lies beyond the scope of the present work.

The DFPT implementation in the HP code of Quantum ESPRESSO is generally

more efficient, scalable, and better suited for automation than the supercell approach. In addition to being substantially more computationally demanding, supercell calculations are more difficult to converge and require symmetry-based specific pre- and post-processings to extract the Hubbard parameters. For these reasons, we focus on improving the robustness of the DFPT workflow rather than falling back to supercell-based methods.

In the systems where failures occurred (e.g., $Br_8Li_4Mn_2$ and $Li_4Mn_2O_8$), the issue was related to technical incompatibilities, such as an incompatible FFT grid with the symmetry operations. These can often be resolved by adjusting input parameters (e.g. the dimensions of the FFT grid), by optimizing the structure better, or even by drastically disabling the use of symmetry. Nevertheless, such interventions are not always desirable from a user-perspective, as these would lead to changing sensible input parameters that can even result in a dramatic increase of the computational cost. In the future, we could implement and make optional such automated recovery strategies in new versions of the `aiida-hubbard` plugin. Suggestions on how to manually address these specific cases will be highlighted in a dedicated troubleshooting section of the presented code.

We finally emphasize that the framework is designed to accommodate alternative schemes for computing Hubbard parameters, including emerging approaches based on orbital resolution [3] or Wannier functions as Hubbard projectors [6].

II. SUPPLEMENTARY FIGURES

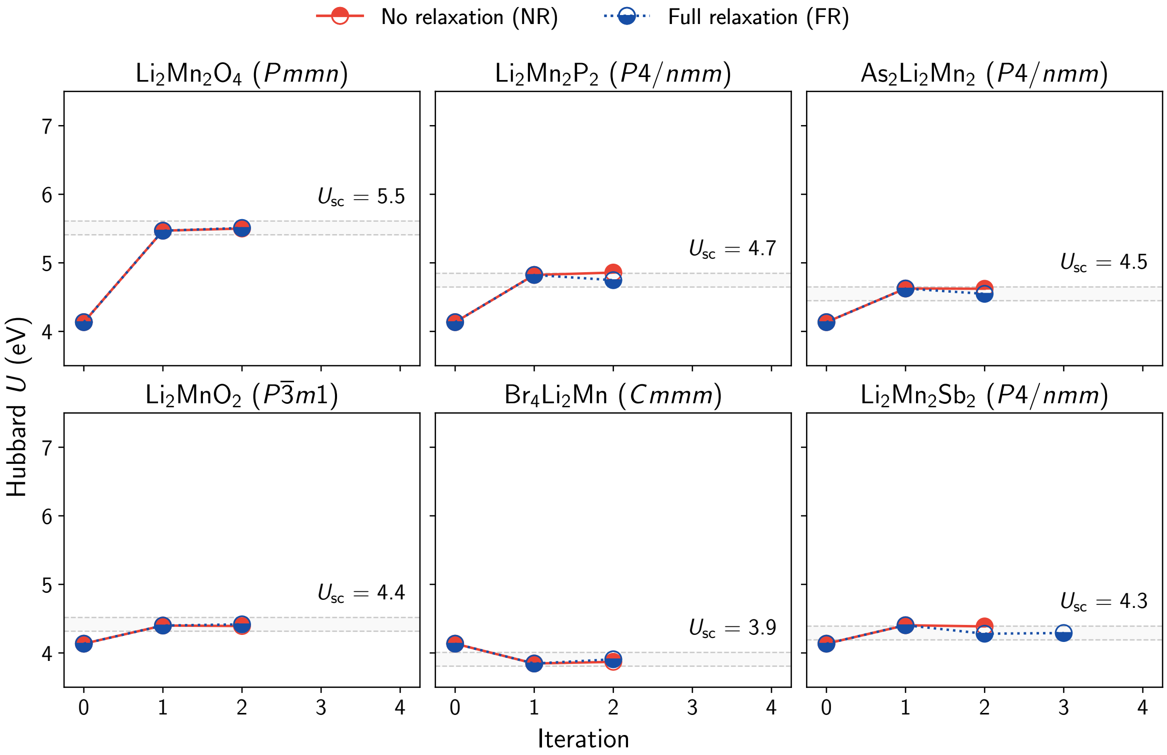

Supplementary Figure 2. **Self-consistent convergence of Hubbard U for Mn-3d using two different strategies.** Values of Hubbard U for six different Mn-containing bulk solids as a function of the iteration of the self-consistent cycle, using the NR (red) and FR (blue) schemes. For each compound, the associated Hill formula and space group are reported in the title of the corresponding panel. Iteration 0 corresponds to the starting guess $U_{\mathrm{Mn}} = 4$ eV. Geometry optimizations of the FR cycle were omitted in iteration 1. The gray shaded area shows the convergence range $U_{\mathrm{sc}} \pm \delta U$ ($\delta U = 0.1$ eV), around the final values of the FR cycles.

III. SUPPLEMENTARY TABLES

Supplementary Table I. **Summary of the protocol main calculation parameters pre-defined by using the `get_builder_from_protocol`.** List of parameters defined for the devised protocols available in the `aiida-hubbard` plugin v0.2.0. Each protocol is identified by a string ("Name"), and it is associated to some pre-definition of calculation and computational resources parameters. Here we report, in order from left to right: the pseudopotentials (kinetic and charge density cutoffs are set according to the associated cutoffs tables) taken from the corresponding SSSP library v1.3 for the PBEsol functional, the **k**-point distance for the DFT+$U+V$ and structural optimization step, the **q**-point distance for the DFTP calculation, and finally the thresholds for the Hubbard parameters U and V determining when the workflow reached self-consistency. Other specific parameters can be found on the corresponding GitHub repositories, by using the Python API of the associated WorkChains, or by inspecting the available online documentation.

Name	SSSP/1.3/PBEsol	$\Delta\mathbf{k}^{\mathrm{DFT}+U+V}$	$\Delta\mathbf{k}^{\mathrm{Relax}}$	$\Delta\mathbf{q}^{\mathrm{DFPT}}$	δU	δV
fast	efficiency	0.60	0.30	1.20	0.20	0.10
balanced	efficiency	0.40	0.15	0.80	0.10	0.01
stringent	precision	0.20	0.10	0.40	0.01	0.005

Supplementary Table II: **Summary of relevant data for the 105 Li-bearing materials.** List of the 105 crystal structures for which the `SelfConsistentHubbardWorkChain` calculation completed successfully. The table reports the formula Hill of the material ("Formula"), its space group ("SG"), the electronic band gap (E_{gap}) computed using the last called `PwBaseWorkChain` calculation of the workflow (0.0 means metallic), the transition metal(s) ("TM") and its oxidation state(s) and computed U_{sc}, the range of computed V_{sc} and associated interatomic distances, the number of V_{sc} interactions defined for the TM atom ($\mathrm{N}_{V_{\mathrm{sc}}}$), the number of atoms inside the unit cell, the first eight digits of the UUID (indicated by UUID*) of the final `HubbardStructureData` associated with the crystal structure. Electronic band gap and Hubbard parameters are given in eV units. The list is ordered by TM first, then by its OS (ascending order), and finally by the numerical value of the associated U_{sc} (ascending order).

Formula	SG	E_{gap}	TM	OS	U_{sc}	V_{sc} (min-max)	r (min-max)	$\mathrm{N}_{V_{\mathrm{sc}}}$	N_{at}	UUID*
$Fe_4Li_4O_{16}P_4$	*Pnma*	4.2	Fe	2	5.23	0.39 – 0.88	2.06 – 2.25	6	28	016fa32c
$As_2Fe_2Li_2$	*P4/nmm*	0.0	Fe	2	5.37	–	–	–	6	13a84d6c
$Ba_4Fe_4Li_2N_6$	*C2/c*	0.0	Fe	2	5.37	–	–	–	16	1e0f3e74
$Fe_2Li_4O_8Si_2$	*Pc*	3.4	Fe	2	5.38	0.5 – 0.64	1.99 – 2.06	4	16	a3142187
$Fe_2Li_2P_2$	*Cmcm*	0.0	Fe	2	5.38	–	–	–	6	b6241509
$Fe_2Li_2P_2$	*P4/nmm*	0.0	Fe	2	5.40	–	–	–	6	9e86853f
$B_4Fe_4Li_4O_{12}$	*C2/c*	3.5	Fe	2	5.40	0.26 – 0.84	1.97 – 2.29	5	24	83466658
$Fe_2Li_4O_8Si_2$	*Pmn*2$_1$	3.5	Fe	2	5.40	0.51 – 0.67	2.0 – 2.07	4	16	dd95032a
$Fe_4Li_8O_{16}Si_4$	*Pnma*	3.5	Fe	2	5.43	0.48 – 0.7	1.99 – 2.12	4	32	e591a01f
$Fe_4Li_8O_{16}Si_4$	*P*2$_1$*/c*	3.5	Fe	2	5.43	0.51 – 0.69	1.99 – 2.09	4	32	467dde85
$F_2Fe_2Li_4O_8P_2$	*P*1	4.2	Fe	2	5.43	0.75 – 0.99	2.05 – 2.11	4	18	fed35928
$Fe_2Li_2O_8P_2$	*Cmcm*	3.9	Fe	2	5.45	0.54 – 0.95	2.06 – 2.19	6	14	f01cfc97
$As_4Fe_4Li_4O_{16}$	*Pnma*	2.3	Fe	2	5.46	0.42 – 0.9	2.07 – 2.27	6	28	5e922c44
$Fe_2Ge_2Li_4O_8$	*Pmn*2$_1$	2.6	Fe	2	5.47	0.57 – 0.74	2.0 – 2.06	4	16	92020bef
$Fe_4Ge_4Li_8O_{16}$	*Pnma*	2.6	Fe	2	5.49	0.56 – 0.77	1.99 – 2.06	4	32	99dc4c84
$Cl_2Fe_2Li_2O_8W_2$	*P*2$_1$*/m*	3.5	Fe	2	5.97	0.72 – 1.24	2.03 – 2.29	4	16	6277cc5e

Continued on next page

Formula	SG	E_{gap}	TM	OS	U_{sc}	V_{sc} (min-max)	r (min-max)	$N_{V_{sc}}$	N_{at}	UUID*
$Fe_2Li_2O_8P_2$	$P\bar{1}$	3.3	Fe	2	6.00	0.67 – 1.01	1.95 – 2.08	4	14	e97dc3bb
$Cl_2Fe_2Li_2Mo_2O_8$	$P2_1/m$	2.9	Fe	2	6.07	0.77 – 1.3	2.03 – 2.28	4	16	3b438081
Fe_6Ge_4Li	$R\bar{3}m$	0.0	Fe	2	6.10	–	–	–	11	aafb22c5
$FeLi_2O_8W_2$	$P\bar{1}$	2.9	Fe	2	6.50	0.78 – 1.15	2.05 – 2.22	6	13	6e4b5fe5
$F_6FeLiRb_2$	$Fm\bar{3}m$	3.3	Fe	3	4.43	–	–	–	10	824cae1e
$F_{12}Fe_2Li_2Rb_4$	$R\bar{3}m$	3.1	Fe	3	4.45	–	–	–	20	1b1931aa
$Ca_2F_{12}Fe_2Li_2$	$P\bar{3}1c$	3.6	Fe	3	4.51	–	–	–	18	09b6f4a3
$F_{18}Fe_3Li_3Mn_3$	$P321$	1.9	Fe<br>Mn	3<br>2	4.54<br>3.60	–	–	–	27	d10e99d6
$Cs_4F_{12}Fe_2KLi$	$R\bar{3}m$	3.6	Fe	3	4.59	–	–	–	20	9592f972
$Cd_2F_{12}Fe_2Li_2$	$P\bar{3}1c$	3.2	Fe	3	4.59	–	–	–	18	41628b5a
$F_{12}Fe_4Li_2$	$P4_2nm$	1.7	Fe	3	4.62	–	–	–	18	0f074e2f
$Br_{16}Fe_4Li_4$	$P2_1/c$	1.6	Fe	3	4.65	–	–	–	24	b2a84fa1
$Cl_{16}Fe_4Li_4$	$P2_1/c$	2.2	Fe	3	4.65	–	–	–	24	d4348d92
$Fe_4Li_4O_8$	$Pna2_1$	1.7	Fe	3	4.88	0.74 – 0.77	1.9 – 1.91	4	16	38c1c476
$Fe_4Li_4O_{16}Si_4$	$Pna2_1$	2.9	Fe	3	4.91	0.8 – 0.85	1.88 – 1.9	4	28	44219f86
$Fe_2Li_2O_8Si_2$	Pc	2.9	Fe	3	4.94	0.76 – 0.84	1.88 – 1.91	4	14	d8392931
$FeLiO_2$	$R\bar{3}m$	1.7	Fe	3	4.97	0.53 – 0.53	2.04 – 2.04	6	4	565f1e2b
FeLiP	$I4mm$	0.0	Fe	3	5.00	–	–	–	3	d13a74d8
AsFeLi	$I4mm$	0.0	Fe	3	5.02	–	–	–	3	53d2fb1a
$As_2Fe_2Li_2$	$P6_3/mmc$	0.0	Fe	3	5.05	–	–	–	6	2d5940ab
$Fe_2Li_2O_4$	$I4_1/amd$	1.0	Fe	3	5.09	0.63 – 0.66	2.02 – 2.05	6	8	ba2f2c65
$C_6Cs_2FeLiN_6$	$Fm\bar{3}m$	1.6	Fe	3	5.09	–	–	–	16	f3174ac0
$Fe_2Li_2O_{12}Si_4$	$C2/c$	2.9	Fe	3	5.10	0.57 – 0.97	1.92 – 2.17	6	20	c68d530e
$Fe_2H_2Li_2O_{10}P_2$	$P\bar{1}$	2.4	Fe	3	5.22	0.71 – 0.99	1.96 – 2.06	6	18	58db6c75
$Fe_2Ge_4Li_2O_{12}$	$C2/c$	2.5	Fe	3	5.22	0.68 – 0.95	1.94 – 2.13	6	20	7fc2690b
$C_{12}Fe_2Li_2N_{12}Rb_4$	$P2_1/c$	1.9	Fe	3	5.22	–	–	–	32	b80935ff

Continued on next page

Formula	SG	E_{gap}	TM	OS	U_{sc}	V_{sc} (min-max)	r (min-max)	$N_{V_{sc}}$	N_{at}	UUID*
$Fe_2Li_2O_{14}P_4$	$P2_1$	3.0	Fe	3	5.41	0.86 – 1.08	1.95 – 2.08	6	22	751f1d3b
As_2FeLiO_7	$C2$	2.6	Fe	3	5.54	0.88 – 1.24	1.94 – 2.05	6	11	9e4f1352
$Fe_2Li_6N_4$	$Ibam$	0.0	Fe	3	5.56	–	–	–	12	afa34d07
$Fe_2Li_2O_{20}Se_8$	$Pnc2$	2.1	Fe	3	5.59	0.82 – 1.17	2.0 – 2.07	6	32	19fd0c67
$FeLi_4N_2$	$Immm$	0.8	Fe	3	5.65	–	–	–	7	9c509aa5
$Fe_2Li_2Mo_4O_{16}$	$P\bar{1}$	2.3	Fe	3	5.73	0.93 – 1.31	1.96 – 2.06	6	24	6999e744
$Fe_2Li_2O_{16}W_4$	$C2/c$	2.5	Fe	3	5.73	1.03 – 1.13	1.98 – 2.09	6	24	b9a97395
Br_4FeLi_2	$Cmmm$	0.0	Fe	3	5.75	–	–	–	7	998cc0b5
$Fe_2Li_4O_{16}P_4$	$P2_1/c$	0.2	Fe	3	5.86	1.07 – 1.37	1.97 – 2.09	6	26	c5c3fa90
$FeLi_2S_2$	$P\bar{3}m1$	0.0	Fe	3	5.93	0.34 – 0.34	2.57 – 2.57	6	5	0d7aaecb
$Fe_2Li_4S_8Sn_2$	Pc	0.0	Fe	3	5.93	0.47 – 0.64	2.37 – 2.39	4	16	7e48fbf6
Cl_8FeLi_6	$Fm\bar{3}m$	0.0	Fe	3	6.21	–	–	–	15	c5abf8a0
$FeLa_2LiO_6$	$R\bar{3}$	0.9	Fe	5	7.12	0.59 – 0.59	1.86 – 1.86	6	10	62db0762
F_8HfLi_2Mn	$I\bar{4}$	5.7	Mn	2	3.58	–	–	–	12	af8b1875
F_8Li_2MnZr	$I\bar{4}$	5.1	Mn	2	3.73	–	–	–	12	24fece8d
$F_{18}Li_3Mn_3Ti_3$	$P321$	4.1	Mn	2	3.75	–	–	–	27	88c37e8a
			Ti	3	5.16					
Br_4Li_2Mn	$Cmmm$	3.5	Mn	2	3.91	–	–	–	7	9004cf7d
$F_{18}Li_3Mn_3V_3$	$P321$	2.8	Mn	2	3.91	–	–	–	27	64e44887
			V	3	4.47					
$Li_8Mn_2Se_{14}Sn_4$	Cc	1.5	Mn	2	4.19	0.12 – 0.13	2.57 – 2.59	4	28	0a8bc9a6
$LiMnTe_2$	$P3m1$	0.0	Mn	2	4.29	0.17 – 0.22	2.67 – 2.75	4	4	78fac50c
$Li_2Mn_2Sb_2$	$P4/nmm$	0.0	Mn	2	4.31	–	–	–	6	dff4cc5c
$Li_4Mn_4O_{16}P_4$	$Pnma$	3.2	Mn	2	4.36	0.38 – 0.47	2.18 – 2.24	6	28	184981a5
$Li_4Mn_2S_8Sn_2$	$Pmn2_1$	1.9	Mn	2	4.38	0.11 – 0.18	2.44 – 2.46	4	16	1eeb5e7d
$Li_8Mn_4S_{16}Sn_4$	$Pna2_1$	2.0	Mn	2	4.43	0.17 – 0.17	2.43 – 2.45	4	32	1b301d2a
$Li_4Mn_2S_8Sn_2$	Pc	2.0	Mn	2	4.44	0.17 – 0.18	2.43 – 2.45	4	16	a5514012

Continued on next page

Formula	SG	E_{gap}	TM	OS	U_{sc}	V_{sc} (min-max)	r (min-max)	$N_{V_{sc}}$	N_{at}	UUID*
$Ge_4Li_8Mn_4S_{16}$	$Pna2_1$	2.1	Mn	2	4.45	0.14 – 0.16	2.42 – 2.45	4	32	fb561bdb
$Ge_4Li_8Mn_2S_{14}$	Cc	2.4	Mn	2	4.45	0.15 – 0.19	2.42 – 2.47	4	28	c03f0795
$Li_2Mn_2O_8$	$I4_1/amd$	0.0	Mn	2	4.56	0.48 – 0.84	2.15 – 2.64	12	12	ce87c8fe
$As_2Li_2Mn_2$	$P4/nmm$	0.0	Mn	2	4.57	–	–	–	6	a085ae77
Li_2MnO_2	$P\bar{3}m1$	2.6	Mn	2	4.57	0.22 – 0.22	2.25 – 2.25	6	5	14989b62
$Li_2Mn_2Na_2O_8Si_2$	Pc	2.7	Mn	2	4.61	0.54 – 0.58	2.05 – 2.07	4	16	78af6e7a
AsLiMn	$F\bar{4}3m$	0.1	Mn	2	4.71	–	–	–	3	25503b48
$Li_2Mn_2O_8P_2$	$Cmcm$	3.3	Mn	2	4.71	0.56 – 0.8	2.13 – 2.21	6	14	004e178f
$As_4Li_4Mn_4O_{16}$	$Pnma$	1.7	Mn	2	4.74	0.42 – 0.81	2.13 – 2.29	6	28	8ce9b10d
$Li_2Mn_2P_2$	$P4/nmm$	0.0	Mn	2	4.74	–	–	–	6	d5c1a4b5
$K_2Li_2Mn_2O_4$	$C2/m$	1.3	Mn	2	4.75	0.24 – 0.46	2.04 – 2.18	4	10	2c4f07e9
$B_3Li_3Mn_3O_9$	$P\bar{6}$	2.7	Mn	2	4.81	0.44 – 0.59	2.08 – 2.17	5	18	b74fe341
$Li_4Mn_2O_8Si_2$	$Pmn2_1$	3.2	Mn	2	4.85	0.48 – 0.54	2.06 – 2.09	4	16	3cace8d4
$Li_8Mn_4O_{16}Si_4$	$Pnma$	3.2	Mn	2	4.85	0.48 – 0.57	2.06 – 2.09	4	32	39ceaf22
$Li_4Mn_2O_8Si_2$	Pc	3.1	Mn	2	4.86	0.47 – 0.58	2.04 – 2.08	4	16	9f68f1d1
$Ge_2Li_4Mn_2O_8$	$Pmn2_1$	2.5	Mn	2	4.90	0.54 – 0.6	2.06 – 2.09	4	16	d747d36d
$LiMnSe_2$	$P3m1$	0.0	Mn	2	4.91	0.31 – 0.4	2.46 – 2.55	4	4	4ea95a4f
$Li_2Mn_2O_8V_2$	$Cmcm$	3.1	Mn	2	5.33	0.64 – 0.75	2.16 – 2.2	6	14	3a23d201
			V	5	5.85	1.25 – 1.28	1.69 – 1.75	4		
$BaLi_2MnO_8V_2$	$P\bar{3}$	3.8	Mn	2	5.31	0.75 – 0.75	2.18 – 2.18	6	14	be9b8a1d
			V	5	5.71	1.34 – 1.38	1.70 – 1.73	4		
$KLiMn_2O_{12}Si_4$	$C2/m$	2.3	Mn	3	5.64	0.4 – 0.78	1.88 – 2.26	6	20	294b9fe4
$F_{10}Li_4Mn_2$	$C2/c$	2.1	Mn	3	5.66	–	–	–	16	c4a9c584
$Li_2Mn_2O_4$	$Pmmn$	1.4	Mn	3	5.75	0.29 – 0.62	1.92 – 2.31	6	8	a2d005d1
$F_8Li_2Mn_2$	$P2_1/c$	2.2	Mn	3	5.79	–	–	–	12	abcf4222
$Li_4Mn_4O_8$	$I4_1/amd$	1.5	Mn	3	5.81	0.29 – 0.63	1.94 – 2.33	6	16	eddfe602
$F_8Li_6Mn_2O_{12}P_4$	$P2_1/c$	2.0	Mn	3	6.08	0.7 – 0.91	1.91 – 2.19	4	32	1f4176f0

Continued on next page

Formula	SG	E_{gap}	TM	OS	U_{sc}	V_{sc} (min-max)	r (min-max)	$N_{V_{sc}}$	N_{at}	UUID*
$H_2Li_2Mn_2O_{10}P_2$	$P\bar{1}$	1.2	Mn	3	6.29	0.64 – 1.03	1.9 – 2.25	6	18	787f2595
$Li_2Mn_2O_{14}P_4$	$P2_1$	1.9	Mn	3	6.34	0.76 – 1.05	1.91 – 2.19	6	22	11150a88
$Ca_2Li_6Mn_2N_6$	$R\bar{3}$	0.7	Mn	4	5.98	–	–	–	16	5df0d47e
$Li_8Mn_4O_{12}$	$C2/c$	2.1	Mn	4	6.49	0.68 – 0.69	1.92 – 1.93	6	24	c78b6e9c
$Li_4Mn_2O_6$	$C2/m$	1.9	Mn	4	6.49	0.68 – 0.69	1.92 – 1.93	6	12	6b53bc4b
$Li_4Mn_4Ni_2O_{12}$	$Cmce$	1.6	Mn	4	6.51	0.6 – 0.61	1.9 – 1.95	6	22	504172a5
			Ni	2	5.16	0.38 – 0.47	2.03 – 2.1			
$Li_2Mn_2O_{10}P_2$	$P\bar{1}$	0.0	Mn	4	7.23	0.94 – 1.47	1.85 – 2.11	6	16	a44df345
$Li_2Mn_4O_8$	$Fd\bar{3}m$	0.0	Mn	4	9.21	1.29 – 1.29	2.01 – 2.01	6	14	76ad08cf
$K_{11}LiMn_4O_{16}$	$I\bar{4}2m$	2.1	Mn	5	5.40	0.63 – 0.65	1.7 – 1.7	4	32	9eb5676a
$Li_6Mn_2O_8$	$Pmn2_1$	2.1	Mn	5	5.80	0.68 – 0.73	1.7 – 1.71	4	16	ad395cda
$Cs_4Li_2Mn_2O_8$	$Cmc2_1$	2.2	Mn	5	5.82	0.63 – 0.73	1.69 – 1.72	4	16	61002306
$Li_{12}Mn_4O_{16}$	$Pnma$	2.1	Mn	5	5.82	0.72 – 0.73	1.7 – 1.71	4	32	d6d86f57
$Li_2Mn_2O_8$	$Cmcm$	1.7	Mn	7	6.55	0.5 – 0.63	1.59 – 1.61	4	12	c745c192

Supplementary Table III: **Information about the 10 failed calculations.** List of the 10 crystal structures for which the `SelfConsistentHubbardWorkChain` calculation stopped and the self-consistent Hubbard parameters have not been computed. The table reports the formula Hill of the material ("Formula"), its space group ("SG"), the calculation step where workflow was interrupted ("Step of failure"), and the more specific associated message describing the reason of the failure ("Exit message").

Formula	SG	Step of failure	Exit message
$As_4Fe_4Li_4$	$P\bar{3}m1$	DFT+$U+V$	The electronic minimization cycle failed during an ionic minimization cycle.
$Fe_2Li_2O_8$	$Fd\bar{3}m$	DFT+$U+V$	The S matrix was found to be not positive definite.
$Br_8Li_2Mn_8$	$I4_1/amd$	DFT+$U+V$	The S matrix was found to be not positive definite.
$As_2Li_2Mn_2O_{10}$	$P\bar{1}$	Structure Optimization	The electronic minimization cycle did not reach self-consistency.
$F_{12}Li_2Mn_2V_2$	$P4_2nm$	Structure Optimization	The stdout output file was incomplete probably because the calculation got interrupted.
F_6FeLi_3	$Fm\bar{3}m$	DFPT	The stdout output file was incomplete probably because the calculation got interrupted.
$Br_8Li_4Mn_2$	$Imma$	DFPT	The code failed due to an incompatible FFT grid.
$Li_4Mn_2O_8$	$Fddd$	DFPT	The code failed due to an incompatible FFT grid.
$C_6Cs_2LiMnN_6$	$Fm\bar{3}m$	Max iteration reached	–
$C_6CuFeLi_2N_6$	$Fm\bar{3}m$	Max iteration reached	–

IV. SUPPLEMENTARY REFERENCES

[1] Kulik, H. J. & Marzari, N. Systematic study of first-row transition-metal diatomic molecules: A self-consistent DFT+U approach. *J. Chem. Phys.* **133**, 114103, DOI: 10.1063/1.3489110 (2010).

[2] Yu, K. & Carter, E. A. Communication: Comparing ab initio methods of obtaining effective U parameters for closed-shell materials. *The J. Chem. Phys.* **140**, DOI: 10.1063/1.4869718 (2014).

[3] Macke, E., Timrov, I., Marzari, N. & Ciacchi, L. C. Orbital-resolved DFT+U for molecules and solids. *J. Chem. Theory Comput.* **20**, 4824–4843, DOI: 10.1021/acs.jctc.3c01403 (2024).

[4] Mariano, L. A., Vlaisavljevich, B. & Poloni, R. Improved Spin-State Energy Differences of Fe(II) Molecular and Crystalline Complexes via the Hubbard U-Corrected Density. *J. Chem. Theory Comput.* **17**, 2807–2816, DOI: 10.1021/acs.jctc.1c00034 (2021).

[5] Cococcioni, M. & de Gironcoli, S. Linear response approach to the calculation of the effective interaction parameters in the LDA+U method. *Phys. Rev. B* **71**, 035105, DOI: 10.1103/physrevb.71.035105 (2005).

[6] Carta, A., Timrov, I., Mlkvik, P., Hampel, A. & Ederer, C. Explicit demonstration of the equivalence between DFT+U and the Hartree-Fock limit of DFT+DMFT. *Phys. Rev. Res.* **7**, 013289, DOI: https://doi.org/10.1103/PhysRevResearch.7.013289 (2025).
